# Supplementary material for: Zika Virus Non-Structural Protein 1 Antigen-Capture Immunoassay
Source: Viruses. 2021 Sep 5;13(9):1771. doi: 10.3390/v13091771 (PMC8473068; doi:10.3390/v13091771)
Supplement: Supplementary file 1 [file viruses-13-01771-s001.zip › viruses-1349734-supplementary.pdf]

SUPPLEMENTAL MATERIALS

Zika Virus Non-Structural Protein 1 Antigen-Capture Immunoassay

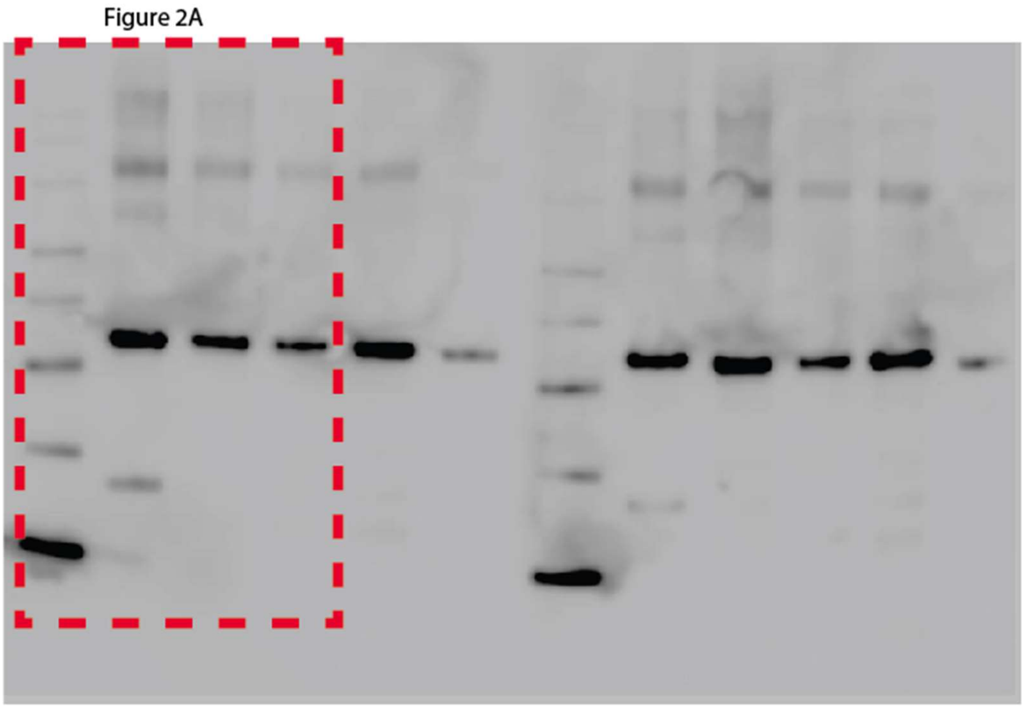

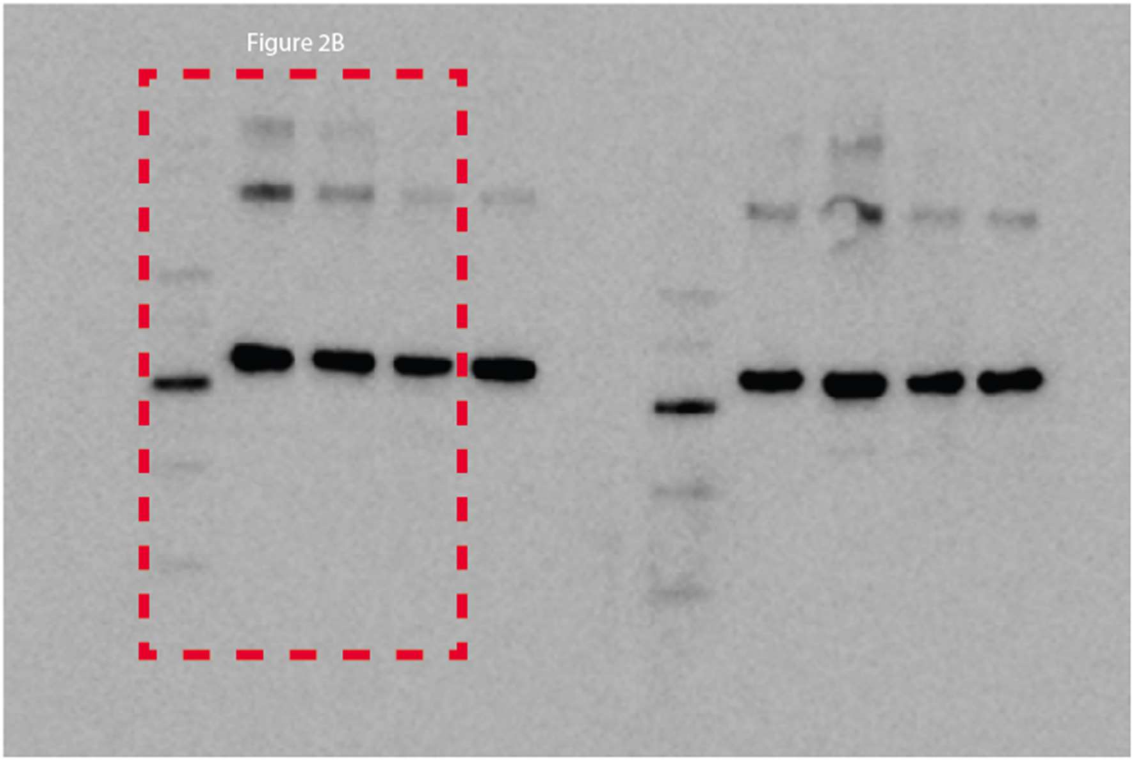

Figure S1. Uncropped gel images for Figure 2.

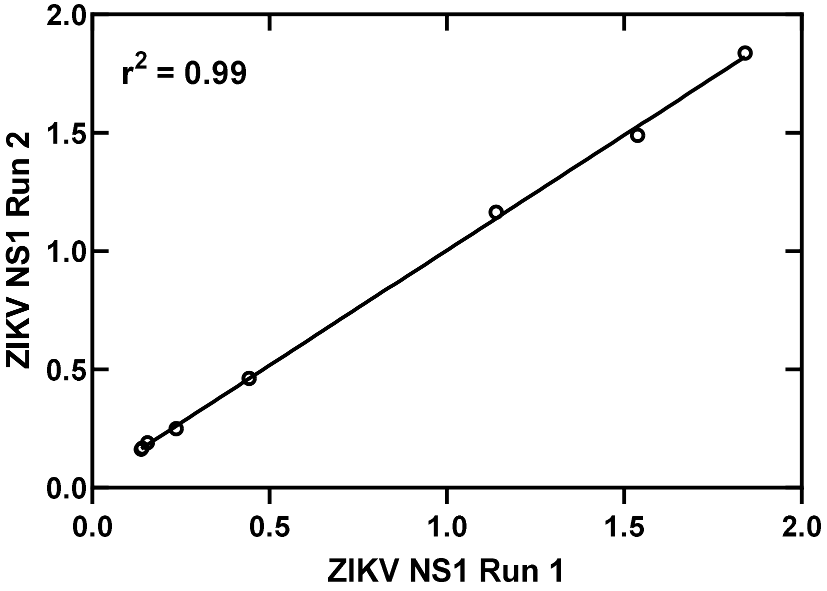

Figure S2. Antigen capture assay shows consistency between runs. Two runs using the same preparation of anti-ZIKV NS1 pAbs. $P < 0.0001$, as determined through Spearman correlation.

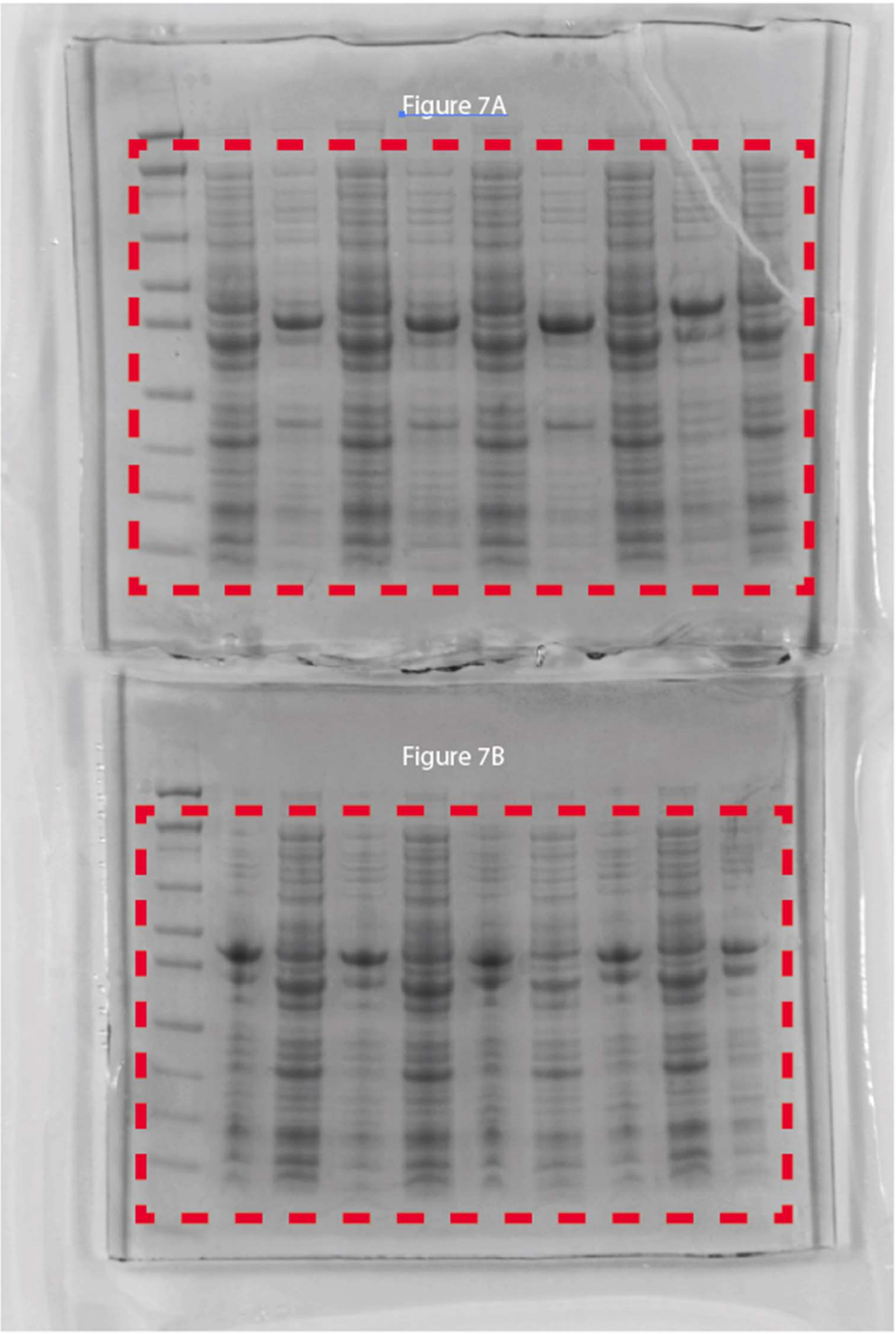

Figure S3. Uncropped gel images for Figure 7.

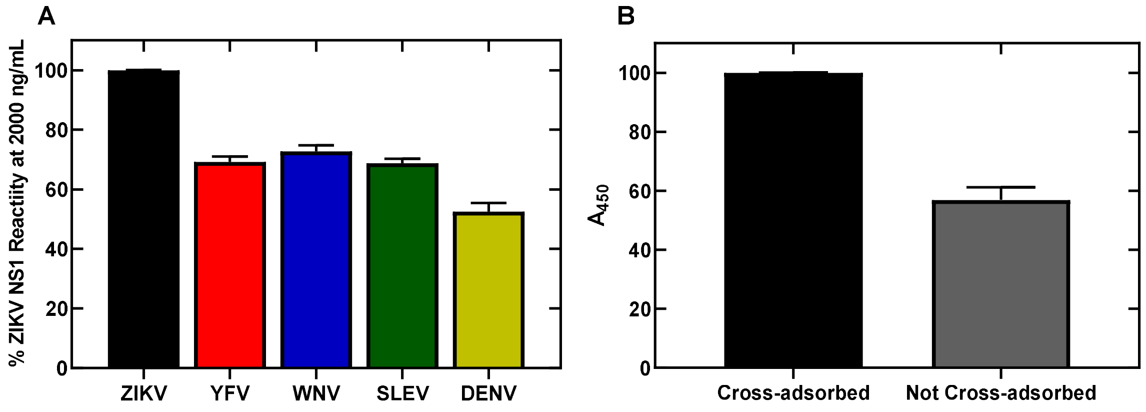

Figure S4. Reactivity of NS1 antibodies without cross-adsorption. (**A**) Related Flavivirus reactivity using NS1 antibodies non-adsorbed against DENV NS1 shows binding at a high percentage of ZIKV NS1 levels. (**B**) Comparison of ZIKV NS1 reactivity using adsorbed and non-adsorbed NS1 antibodies shows higher signal intensities of cross-adsorbed antibodies to ZIKV NS1 protein.

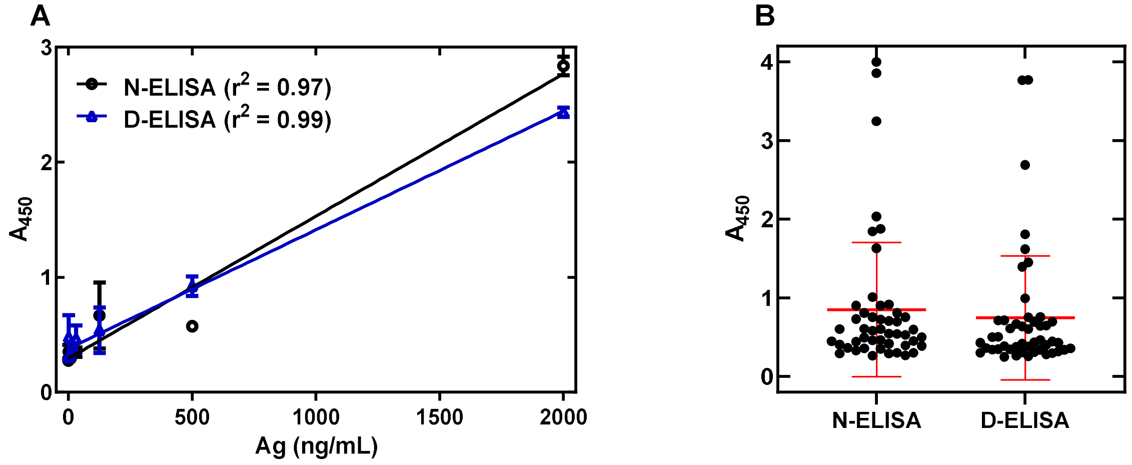

Figure S5. ZIKV NS1 Capture ELISA does not display interference from substances in patient serum. Antibodies against NS1 were dissociated from antigen via detergent and assayed for increased activity using the antigen capture ELISA. (A) The assay was still able to detect ZIKV NS1 well in both N-ELISA (dissociation solution was neutralized before addition of NS1) and D-ELISA (dissociation solution was neutralized in the presence of NS1). (B) No significant difference was seen in ZIKV NS1 detection in patient samples between N-ELISA and D-ELISA conditions as determined by Mann-Whitney test.

Table S1. Primer sequences used for the generation and mutation of ZIKV/DENV NS1.

Primer Designation	Primer Sequence
ZIKV NS1 WT F	5′-ATCGATGGATCCCGTGGGGTGCTCAGTGGAC-3′
ZIKV NS1 WT R	5′-ATCGATAAGCTTTTATGACCCCGCTGTCACCA-3′
DENV2 NS1 WT F	5′-ATCGATGGATCCCGATAGTGGTTGCGTTGTG-3′
DENV2 NS1 WT R	5′-ATCGATGCGGCCGCCTTAGGCTGTGACCAAGGAGC-3′
117, 118, 119-AAA F	5′-ATGAGCTGCCCCATGGCTGGAAAGCCGCTGCAGCATCGTATTTTGTTAGG-3′
117, 118, 119-AAA R	5′-CTGTTGTTGGTCTTTGCCGCCCTAACAAAATACGATGCTGCAGCGGCTTTC CAGCCG-3′
227, 228, 229-AAA F	5′-GAATGGCCAAAGTCTGCCGCTGCCTGGACAGATGGAGTAGAAGAAAGTGATCTTATCATACCC-3′
227, 228, 229-AAA R	5′-CTCCATCTGTCCAGGCAGCGGCAGACTTTGGCCATTCACATGTTTTCATCTCAATCAGGTG-3′

Table S2. Primers used for the Generation of Flavivirus NS1 Proteins.

Primer Designation	Primer Sequence
SLEV NS1 WT F	5′-ATCGATGGATCCCGCTGATTCGGGATGTGCA-3′
SLEV NS1 WT R	5′-ATCGATAAGCTTTTAAGCTGTCACTCGAGATTT-3′
WNV NS1 WT F	5′-ATCGATGGATCCCGACACTGGGTGTGCCATA-3′
WNV NS1 WT R	5′-ATCGATGCGGCCGCTTAAGCATTCACTTGTGACTG-3′
YFV NS1 WT F	5′-ATCGATCAATTGCCAAGGATGCGCCATCAAC-3′
YFV NS1 WT R	5′-ATCGATGCGGCCGCTTATATTTCTCCAGCTGTAAC-3′

Table S3. NS1/IgM/IgG Levels in Surveyed Patient Serum. Sample ID numbers beginning with Z originate from Colombia, while those beginning with DR originate from the Dominican Republic.

Sample ID	IgM WT	IgM 117-119	IgM 227-229	IgG WT	IgG 117-119	IgG 227-229	NS1 Concentration (ng/mL)
**Z-10**	0.18675	0.13835	0.12285	1.20945	1.11725	1.14445	82.05
**Z-100**	0.18045	0.12485	0.12275	0.59875	0.7307	0.61055	83.98
**Z-101**	0.1868	0.1559	0.10545	1.88775	1.86325	1.82465	ND
**Z-102**	0.1221	0.1034	0.0889	1.41885	1.4985	1.4237	221.8
**Z-103**	0.5427	0.34535	0.1813	0.12735	0.0831	0.07875	73.8
**Z-104**	0.19075	0.1023	0.0616	0.4231	0.4824	0.4387	11.68
**Z-105**	0.3016	0.19225	0.12755	1.77325	1.7653	1.76545	10.48
**Z-106**	0.5318	0.31145	0.1454	1.05435	1.18865	1.1215	60.99
**Z-107**	0.1959	0.10525	0.0644	0.8615	1.04225	0.93125	54.11
**Z-110**	0.35795	0.2078	0.09665	1.78745	1.78155	1.7858	625.48
**Z-115**	0.18385	0.1088	0.04575	1.86325	1.82935	1.6451	354.03
**Z-116**	0.1003	0.0811	0.07025	1.9695	2.05525	1.97125	50.31
**Z-117**	0.11065	0.07645	0.05305	1.9428	1.92395	1.95725	ND
**Z-118**	0.0924	0.08855	0.0584	1.88995	1.87645	1.8721	36.34
**Z-119**	0.2128	0.14365	0.1116	1.7582	1.75715	1.7436	ND
**Z-12**	0.1454	0.10885	0.17605	2.13385	1.99755	2.00915	ND
**Z-120**	0.5909	0.8051	0.55625	1.6434	1.65535	1.632	72.17
**Z-121**	0.1013	0.09255	0.05955	1.7243	1.7405	1.69705	81.26
**Z-122**	0.26625	0.1755	0.11025	1.46965	1.56365	1.5706	101.9
**Z-123**	0.32225	0.2368	0.1847	1.9463	1.983	1.97375	ND
**Z-124**	0.10315	0.0859	0.0675	1.85715	1.8266	2.00635	17.93
**Z-125**	0.10775	0.09165	0.0798	0.14495	0.13275	0.11665	ND
**Z-126**	0.59	1.13835	1.14065	1.8974	1.90265	1.86705	ND
**Z-127**	0.28025	0.341	0.3252	1.9503	1.9229	1.84155	21.25
**Z-128**	0.2137	0.1842	0.14925	1.77175	1.71435	1.59895	ND
**Z-129**	0.335	0.29555	0.2833	1.4611	1.5747	1.4368	ND
**Z-13**	0.1848	0.12995	0.1248	1.79835	1.73535	1.7803	ND
**Z-130**	0.2383	0.1407	0.39475	0.9602	1.1003	0.95095	88.2
**Z-131**	0.15395	0.1328	0.1037	2.00515	1.896	1.9032	219.51
**Z-132**	0.13245	0.1287	0.11295	2.0958	1.95755	2.0976	ND
**Z-133**	0.1442	0.12745	0.1121	1.83025	1.8161	1.8482	ND
**Z-134**	0.1425	0.1176	0.0942	0.7335	0.636	0.5691	233.32
**Z-135**	0.10035	0.0925	0.06955	1.9588	1.91895	1.90325	ND
**Z-136**	0.29165	0.27365	0.26655	1.4938	1.53255	1.38375	ND
**Z-137**	0.1748	0.1166	0.09645	0.73785	0.77865	0.6258	36.02
**Z-138**	0.2014	0.1909	0.2081	0.21675	0.2691	0.19655	ND
**Z-139**	0.2412	0.1951	0.1497	1.87225	1.8687	1.84365	ND

**Z-14**	0.2553	0.24155	0.2261	1.9442	1.86725	1.95525	–
**Z-140**	–	–	–	–	–	–	269.39
**Z-141**	0.2237	0.1593	0.1923	1.0021	1.24345	1.29265	58.7
**Z-142**	0.245	0.2791	0.27705	0.28615	0.34375	0.2863	ND
**Z-143**	0.42235	0.274	0.22865	1.65795	1.717	1.6307	387.84
**Z-144**	0.1385	0.1042	0.07425	2.01175	1.94975	1.85175	ND
**Z-145**	0.50845	0.44485	0.40785	1.88245	1.8902	1.81805	ND
**Z-146**	0.3082	0.20185	0.11865	1.7067	1.825	1.723	ND
**Z-147**	0.07735	0.0752	0.0489	1.30535	1.3551	1.3287	ND
**Z-148**	0.3067	0.2744	0.24635	1.8125	1.76145	1.85785	30.3
**Z-149**	0.28285	0.24735	0.19545	1.97425	1.93575	2.00455	84.92
**Z-150**	0.2269	0.1854	0.12305	1.8652	1.8393	1.8893	ND
**Z-151**	0.27795	0.2344	0.1948	1.911	1.9248	1.88705	ND
**Z-152**	0.28935	0.24915	0.2001	1.483	1.6654	1.4719	ND
**Z-153**	0.3201	0.24765	0.23835	1.55675	1.7059	1.6278	ND
**Z-154**	0.0911	0.0858	0.07965	1.85525	1.97365	1.89725	ND
**Z-155**	0.2276	0.1663	0.1441	1.75755	1.87555	1.79605	2224.9
**Z-157**	0.0973	0.0973	0.0583	2.0347	2.0291	1.88515	1970.42
**Z-158**	0.29395	0.29395	0.16985	1.96405	1.91655	1.76825	215.39
**Z-159**	0.10485	0.10485	0.05615	0.16	0.13385	0.10675	29.22
**Z-16**	0.4187	0.2703	0.33215	2.0692	1.94725	2.06395	45.99
**Z-160**	0.1442	0.1442	0.0574	1.55275	1.5792	1.4184	131.56
**Z-161**	0.3592	0.3592	0.12335	2.04005	1.9368	1.811	311.05
**Z-162**	0.3122	0.3122	0.1169	0.8183	0.78715	0.6504	ND
**Z-163**	0.22695	0.22695	0.13395	1.4193	1.4626	1.2019	ND
**Z-164**	0.4344	0.4344	0.22625	0.9534	0.9789	0.7495	136.43
**Z-165**	0.3721	0.3721	0.21275	1.9818	1.9589	1.80145	110.54
**Z-166**	0.4125	0.4125	0.25475	1.72945	1.72055	1.4958	ND
**Z-167**	0.2901	0.2901	0.20045	1.8583	1.85295	1.80355	ND
**Z-168**	0.10585	0.10585	0.07455	0.4654	0.4438	0.7577	ND
**Z-169**	0.36165	0.36165	0.17725	1.10865	1.06755	1.15185	75.23
**Z-17**	0.347	0.19665	0.20805	1.37585	1.1515	1.316	104.32
**Z-170**	0.31445	0.31445	0.2202	0.20205	0.1584	0.9291	80.62
**Z-171**	0.25225	0.25225	0.15275	1.4979	1.53325	1.4431	123.14
**Z-172**	0.25345	0.25345	0.14675	1.7365	1.73045	1.39885	44.95
**Z-173**	0.1864	0.1864	0.0476	1.88295	1.8239	1.77385	ND
**Z-174**	0.31205	0.31205	0.1711	1.4173	1.40435	1.36255	30.32
**Z-175**	0.133	0.133	0.1222	1.80045	1.8371	1.816	75.87
**Z-176**	0.07065	0.07065	0.04755	1.19785	1.22845	1.2073	ND
**Z-177**	0.11585	0.11585	0.06905	1.51245	1.5014	1.446	393.65
**Z-178**	0.3342	0.3342	0.2245	1.97355	1.9264	1.83475	99.65

**Z-179**	0.1483	0.1483	0.0924	1.88545	1.8379	1.68495	ND
**Z-18**	0.82585	0.8989	0.8764	2.17995	2.08175	2.1593	ND
**Z-180**	0.20385	0.20385	0.1001	1.5866	1.59005	1.3541	ND
**Z-181**	0.21545	0.21545	0.1011	1.79655	1.70395	1.70775	154.22
**Z-182**	0.2531	0.2531	0.77915	1.96735	1.90445	1.86915	76.67
**Z-183**	0.1187	0.1187	0.0737	1.8534	1.81235	1.8609	290.05
**Z-185**	0.11005	0.11005	0.0667	0.48025	0.4041	0.4594	ND
**Z-187**	0.11115	0.11115	0.076	2.07605	1.94445	1.90215	426.76
**Z-188**	0.29945	0.29945	0.14505	2.02945	1.8909	1.85105	148.28
**Z-189**	0.1754	0.1754	0.06205	1.7437	1.59945	1.58855	88.93
**Z-19**	0.28355	0.19635	0.2093	0.40865	0.30955	0.32515	ND
**Z-191**	0.23635	0.23635	0.16105	2.16735	2.02285	1.96965	81.34
**Z-192**	0.42055	0.3672	0.3458	0.6123	0.8245	0.8703	128.7
**Z-193**	0.13585	0.09715	0.09015	2.04405	2.153	2.0501	ND
**Z-194**	0.19975	0.1472	0.15495	0.27455	0.2828	0.25855	974.72
**Z-195**	0.7876	0.6972	0.7514	1.44715	1.6799	1.6835	58.36
**Z-196**	0.2498	0.1925	0.17915	0.98695	1.25535	1.23515	ND
**Z-197**	0.4337	0.2904	0.3699	0.9509	1.1214	1.0011	76.86
**Z-198**	0.24885	0.20025	0.20225	0.8044	0.87015	0.85485	ND
**Z-199**	0.27275	0.26885	0.2395	2.0184	2.13165	2.0367	64.69
**Z-20**	0.20355	0.1613	0.17315	1.69	1.6782	1.6789	ND
**Z-200**	–	–	–	–	–	–	1277.94
**Z-202**	0.4921	0.50295	0.5169	1.92995	2.0421	2.01765	ND
**Z-21**	0.64465	0.55325	0.49085	0.8335	0.85725	0.8142	ND
**Z-22**	0.3695	0.28635	0.2406	1.9451	1.8883	1.8351	ND
**Z-23**	0.25055	0.19355	0.17165	1.9541	1.86585	1.876	ND
**Z-237**	0.31225	0.15925	0.1819	0.20945	0.1734	0.17695	–
**Z-238**	0.1825	0.1115	0.11135	0.48935	0.59735	0.6182	–
**Z-239**	0.1682	0.0951	0.10215	0.38095	0.41435	0.4214	–
**Z-24**	0.2169	0.16275	0.1614	2.006	1.88895	1.9052	554.32
**Z-240**	0.1566	0.10155	0.10785	0.36965	0.392	0.4085	–
**Z-241**	0.30035	0.21315	0.22715	0.6306	0.74915	0.7864	–
**Z-242**	0.19815	0.14335	0.1403	0.4009	0.66125	0.7135	–
**Z-243**	0.1527	0.08845	0.0845	0.41205	0.36935	0.35185	–
**Z-244**	0.2309	0.1578	0.15525	0.4848	0.4492	0.48665	–
**Z-245**	0.1382	0.0975	0.09775	0.24245	0.22545	0.2328	–
**Z-246**	0.09275	0.0592	0.06615	0.22445	0.2415	0.23645	–
**Z-247**	0.2952	0.27395	0.27515	0.4086	0.4947	0.5101	–
**Z-248**	0.1991	0.133	0.1341	0.7241	0.732	0.75715	–
**Z-249**	0.148	0.10925	0.11235	0.33705	0.34745	0.3316	–
**Z-25**	0.19095	0.1324	0.12165	1.9869	1.89005	1.923	ND

**Z-250**	0.5324	0.34425	0.3553	0.3516	0.42515	0.4041	–
**Z-251**	0.09235	0.0899	0.07505	0.78945	0.84745	0.8697	–
**Z-252**	0.18625	0.12565	0.1345	0.41425	0.51015	0.5498	–
**Z-253**	0.12585	0.07495	0.0855	0.43485	0.5486	0.57155	–
**Z-254**	0.35375	0.1805	0.20305	0.3387	0.3844	0.40415	–
**Z-255**	0.078	0.0602	0.0692	0.5039	0.60715	0.64475	–
**Z-256**	0.12205	0.12705	0.1374	1.53605	1.6347	1.7081	–
**Z-257**	0.11355	0.0805	0.08995	0.0689	0.0606	0.0566	–
**Z-258**	0.11525	0.09205	0.09775	0.28385	0.2433	0.22905	–
**Z-259**	0.1749	0.14505	0.19065	0.23905	0.2813	0.2836	–
**Z-26**	0.15945	0.1306	0.13065	1.9216	1.788	1.78975	ND
**Z-260**	0.33095	0.2642	0.30345	0.62425	0.6791	0.7911	–
**Z-261**	0.4041	0.3056	0.33405	0.7203	0.82835	0.54735	–
**Z-262**	0.21545	0.14235	0.1518	0.4352	0.498	0.5098	–
**Z-263**	0.2176	0.20705	0.17805	0.5991	0.66525	0.68235	–
**Z-27**	0.15365	0.12485	0.1293	1.84135	1.8062	1.7312	ND
**Z-34**	0.2789	0.20765	0.19255	1.8773	1.79885	1.6591	365.27
**Z-35**	0.22015	0.16155	0.1375	1.0025	0.9052	0.71785	POS
**Z-36**	0.14855	0.1264	0.1194	2.1073	1.9994	1.8656	365.27
**Z-37**	–	–	–	–	–	–	ND
**Z-38**	0.1719	0.16195	0.14875	2.10595	2.0817	1.96995	ND
**Z-39**	0.16675	0.1427	0.1192	2.09365	1.9209	1.7449	ND
**Z-40**	0.17235	0.16625	0.14095	1.48305	1.4563	1.41175	ND
**Z-41**	0.3676	0.29905	0.2539	2.14975	2.1056	2.0478	ND
**Z-42**	0.67045	0.66385	0.7727	2.0505	1.95535	1.89305	465.1
**Z-43**	0.1818	0.1472	0.14725	2.0818	2.10905	2.0259	91.12
**Z-44**	0.4155	0.4032	0.3232	2.3109	2.2325	2.17765	ND
**Z-45**	0.48665	0.3742	0.34065	0.45845	0.4349	0.31215	201.6
**Z-46**	0.2071	0.16555	0.1741	1.8365	1.85515	1.67285	ND
**Z-47**	0.15955	0.15785	0.1416	2.1065	2.0067	1.88715	360.25
**Z-48**	0.24085	0.22315	0.23305	1.2557	1.44425	1.52445	123.75
**Z-49**	0.11125	0.10595	0.1159	1.98955	2.0442	2.072	ND
**Z-5**	0.29525	0.17095	0.17285	1.65425	1.35015	1.66575	ND
**Z-50**	0.1386	0.11185	0.1124	0.43375	0.46675	0.44475	ND
**Z-51**	0.1436	0.1051	0.11255	1.29315	1.31895	1.3803	ND
**Z-52**	0.21265	0.1838	0.19555	1.44415	1.70945	1.83175	ND
**Z-53**	0.18045	0.17015	0.1329	0.1907	0.18475	0.1806	51.47
**Z-54**	0.0749	0.09405	0.08075	2.09555	2.05075	2.02425	76.16
**Z-55**	0.22445	0.25075	0.2255	1.71515	1.78425	1.79875	27.05
**Z-56**	0.20445	0.19705	0.1481	1.82595	1.8049	1.8126	254.81
**Z-57**	0.15025	0.1468	0.1024	2.07765	2.03085	1.97875	ND

**Z-58**	0.15925	0.17075	0.1391	2.03995	2.0355	1.94085	469.02
**Z-59**	0.1048	0.09575	0.0707	2.08135	2.0572	1.99555	108.6
**Z-6**	0.19395	0.1413	0.1339	2.107	1.9163	2.0794	370.44
**Z-60**	0.2042	0.17785	0.1629	1.7426	1.7212	1.6583	ND
**Z-61**	0.2194	0.23165	0.16095	0.47285	0.56115	0.64465	224.81
**Z-62**	0.1419	0.144	0.1157	2.07165	2.0312	1.98715	152.12
**Z-63**	0.25645	0.20925	0.15845	2.10235	2.0378	2.04585	ND
**Z-64**	0.1291	0.12755	0.0963	1.6099	1.65585	1.7214	ND
**Z-66**	0.2315	0.2182	0.15595	1.7724	1.7806	1.8385	ND
**Z-67**	0.16785	0.17585	0.1531	2.0853	2.0432	2.01585	ND
**Z-68**	0.21665	0.20305	0.1379	2.09215	2.0181	1.9697	ND
**Z-69**	0.31725	0.2699	0.2126	2.21645	2.26165	2.1225	ND
**Z-70**	0.32695	0.3293	0.2473	2.1993	2.11595	2.0914	ND
**Z-71**	0.2809	0.28905	0.21715	1.77255	1.82185	1.8841	ND
**Z-72**	0.0637	0.0516	0.059	0.94465	1.10855	1.27405	36.16
**Z-73**	0.31675	0.2257	0.22825	1.6721	1.73375	1.7611	49.1
**Z-74**	0.56095	0.59825	0.6439	1.5507	1.5711	1.6324	38.05
**Z-75**	0.36255	0.2715	0.25755	1.504	1.5071	1.5382	658.88
**Z-76**	0.1216	0.1092	0.09945	1.96815	1.91585	1.887	49.11
**Z-77**	0.1078	0.1047	0.0757	0.22335	0.1913	0.1759	158.09
**Z-78**	0.2708	0.2242	0.1825	2.20895	2.05345	2.0541	122.35
**Z-79**	0.0883	0.07305	0.0625	2.0178	1.959	1.9714	ND
**Z-8**	0.1418	0.09255	0.0989	1.6074	1.45935	1.5757	ND
**Z-80**	0.44495	0.38285	0.35135	0.61575	0.5669	0.64285	2112.88
**Z-81**	0.22855	0.18065	0.1472	1.6462	1.60735	1.7455	POS
**Z-82**	0.61205	0.4922	0.50405	0.5077	0.47085	0.49835	1008.49
**Z-83**	0.2784	0.195	0.2174	0.25675	0.23915	0.26535	137.49
**Z-84**	0.1354	0.09125	0.09075	0.65685	0.4402	0.6209	50.1
**Z-85**	0.1751	0.1282	0.1181	1.57815	1.548	1.59465	ND
**Z-86**	0.201	0.13375	0.134	0.89355	0.4379	0.43415	ND
**Z-87**	0.31065	0.25375	0.2445	1.75145	1.7602	1.78615	ND
**Z-88**	0.22535	0.147	0.14695	1.76875	1.72965	1.7167	3184.7
**Z-89**	0.0649	0.0559	0.0519	1.77665	1.7198	1.7254	20.77
**Z-9**	0.2573	0.16085	0.1561	2.0518	1.97985	1.96435	433.98
**Z-90**	0.17595	0.11085	0.078	1.591	1.59325	1.61665	182.59
**Z-91**	0.51245	0.3611	0.28335	1.4643	1.5779	1.47095	495.49
**Z-92**	0.1176	0.10955	0.09285	1.5224	1.5916	1.52355	ND
**Z-93**	0.0769	0.0652	0.05905	1.5744	1.59625	1.46795	ND
**Z-94**	0.15855	0.11015	0.0966	1.49695	1.6053	1.5803	24.22
**Z-95**	0.46565	0.3215	0.2407	1.2856	1.41575	1.36525	905.43
**Z-96**	0.1444	0.1057	0.0945	1.5691	1.66045	1.62075	ND

**Z-97**	0.49495	0.53765	0.49565	1.8198	1.78635	1.84395	304.61
**Z-98**	0.3182	0.20135	0.1508	1.6306	1.6692	1.5995	ND
**Z-99**	0.7843	0.97365	0.78625	1.54175	1.6126	1.53885	55.92
DR 1	0.022	0.024	0.023	0.284	0.409	0.3765	30.47
**DR 2**	0.098	0.095	0.096	0.763	0.7895	0.726	–
**DR 4**	0.043	0.056	0.058	0.035	0.0285	0.028	ND
**DR 7**	–	–	–	–	–	–	54.91
**DR 8**	0.036	0.052	0.049	0.0265	0.023	0.0105	91.3
**DR 9**	–	–	–	–	–	–	20.43
**DR 10**	0.0455	0.0375	0.042	0.669	0.772	0.7405	ND
**DR 11**	0.0595	0.0875	0.0835	0.096	0.1285	0.0945	5.71
**DR 12**	0.013	0.0195	0.0185	0.241	0.2285	0.1585	ND
**DR 13**	0.048	0.056	0.0525	0.5825	0.737	0.6345	66.81
**DR 14**	0.116	0.123	0.14	0.131	0.1625	0.1285	173.48
**DR 15**	0.0805	0.0805	0.0785	0.048	0.066	0.052	83.24
**DR 17**	0.0635	0.0845	0.08	1.253	1.2075	1.103	31.91
**DR 18**	0.067	0.071	0.0685	0.053	0.23	0.102	83.24
**DR 19**	0.055	0.0675	0.0655	0.393	0.495	0.465	ND
**DR 20**	0.068	0.0715	0.071	1.2845	1.1915	0.878	2.37
**DR 21**	0.047	0.066	0.071	0.1	0.1575	0.076	20.64
**DR 22**	0.0645	0.0765	0.074	0.364	0.386	0.2585	ND
**DR 23**	0.1195	0.1535	0.147	0.2815	0.388	0.2565	30.27
**DR 24**	0.1055	0.121	0.116	0.218	0.355	0.259	34.18
**DR 25**	0.0185	0.031	0.0305	0.0615	0.0735	0.054	ND
**DR 26**	0.053	0.062	0.064	0.95	1.038	0.575	ND
**DR 27**	0.0365	0.0425	0.043	0.0295	0.0235	0.0205	ND
**DR 28**	0.053	0.075	0.074	0.044	0.033	0.013	73.3
**DR 29**	0.0835	0.0945	0.0955	0.06	0.0855	0.0305	ND
**DR 30**	0.099	0.108	0.103	0.1915	0.162	0.077	9.26
**DR 31**	0.022	0.024	0.0245	0.042	0.055	0.0235	23.81
**DR 32**	0.027	0.039	0.0425	0.294	0.48	0.2655	ND
**DR 33**	0.0605	0.0705	0.0685	0.327	0.4385	0.257	ND
**DR 34**	0.1805	0.178	0.1735	0.99	0.925	0.4695	115.73
**DR 35**	0.115	0.12	0.1275	0.2825	0.5005	0.4775	4.97
**DR 36**	0.0375	0.064	0.038	0.233	0.308	0.1095	358.6
**DR 37**	0.1075	0.155	0.156	0.179	0.199	0.075	169.11
**DR 38**	0.0475	0.0625	0.0615	0.4125	0.3965	0.1735	1171.01
**DR 39**	0.06	0.086	0.084	0.414	0.5475	0.2575	65.48
**DR 40**	0.036	0.0525	0.056	0.0335	0.022	0.008	157.99
**DR 41**	0.1435	0.16	0.1485	0.047	0.0685	0.024	264.38

POS: Positive, but below limit of quantification. DR: Dominican Republic. 117-119= ZIKV NS1 W117A, G118A, K119A. 227-229= ZIKV NS1 H227A, T228A, L229A.
